# Network Pharmacology Integrated Molecular Docking Reveals the Antiosteosarcoma Mechanism of Biochanin A

**DOI:** 10.1155/2019/1410495

**Published:** 2019-01-06

**Authors:** Qing Luo, Xuan Shi, Jiarong Ding, Zhenzhen Ma, Xumei Chen, Yuanxiu Leng, Xuhui Zhang, Yang Liu

**Affiliations:** ^1^Department of Oncology Laboratory, Affiliated Hospital of Zunyi Medical University, Zunyi, Guizhou, China; ^2^Department of Anaesthesiology, Shanghai Pulmonary Hospital, Tongji University, Shanghai, China; ^3^Department of Nephrology, Shanghai Changhai Hospital, Shanghai, China; ^4^Department of Orthopedics, No. 371 Central Hospital of PLA, Xinxiang, Henan, China

## Abstract

**Background:**

As the malignant tumor with the highest incidence in teenagers, osteosarcoma has become a major problem in oncology research. In addition to surgical management, the pharmacotherapeutic strategy for osteosarcoma treatment is an attractive way to explore. It has been demonstrated that biochanin A has an antitumor capacity on multiple kinds of solid tumor, including osteosarcoma. But the precise mechanism of biochanin A against osteosarcoma is still needed to be discovered.

**Objective:**

To identify the potential therapeutic targets of biochanin A in treating osteosarcoma.

**Methods:**

In present study, an integrated approach including network pharmacology and molecular docking technique was conducted, which mainly comprises target prediction, network construction, gene ontology, and pathway enrichment. CCK8 test was employed to evaluate the cell viability of MG63 cells. Western-blot was used to verify the target proteins of biochanin A.

**Results:**

Ninety-six and 114 proteins were obtained as the targets of biochanin A and osteosarcoma, respectively. TP53, IGF1, JUN, BGLAP, ATM, MAPK1, ATF3, H2AFX, BAX, CDKN2A, and EGF were identified as the potential targets of biochanin A against osteosarcoma. Based on the western-blot detection, the expression of BGLAP, BAX, and ATF3 in MG63 cell line changed under the treatment of biochanin A.

**Conclusion:**

Biochanin A can effectively suppress the proliferation of osteosarcoma and regulate the expression of BGLAP, BAX, and ATF3, which may act as the potential therapeutic targets of osteosarcoma.

## 1. Introduction

Biochanin A is an O-methylated isoflavone and is present in* chickpea, red clover, alfalfa, and cabbage. *Recent reports have mentioned that biochanin A can play a role as a protective factor against osteoporosis, cancers, virus infection, and inflammation, based on its estrogen-like effect, which is also classified as phytoestrogen [1–4]. Along with reports of the antitumor effect of phytoestrogens, biochanin A has also been demonstrated to have an antitumor effect on pharynx squamous carcinoma, hepatocellular carcinoma, pancreatic cancer, prostate cancer, and colon malignancy [2, 5–8].

Osteosarcoma is the most common primary sarcoma of bone in children and young adults with an incidence of 4.4 per million in 0-24-year-old population [9]. Since 80% of osteosarcoma patients have metastatic or micrometastatic diseases at diagnosis, the treatments of osteosarcoma patients are always complicated and few clinical methods can reach the remission [9, 10]. The overall 5-year cumulative survival rate of limb salvage surgery and amputation was 14.6%. Therefore, the chemotherapy is getting more important in the remission of osteosarcoma. Encouragingly, latest research reveals that biochanin A could repress osteosarcoma though regulating cell proliferation, apoptosis, invasion, and migration [11]. However, which precise mechanism and related signal pathways are involved in this process is still unknown. In order to reveal the comprehensive mechanism of biochanin A's antitumor effect, new integrated method is needed, whereas the combination of Omics costs so much.

Network pharmacology is a classic bioinformatics' method, which was conducted to discover the underneath mechanism between drugs and known targets [12]. In the latest years, network pharmacology has been employed to predict the potent targets and pathways of a certain compound in several diseases, especially in the cancer which has an intimate association with signal transduction [12, 13]. Based on the algorithm of network pharmacology, the targets of biochanin A and pathways related to osteosarcoma were analyzed comprehensively in present study, in order to identify the potential therapeutic target and demonstrate the mechanism of biochanin A in treating osteosarcoma.

## 2. Material and Methods

### 2.1. Target Proteins of Biochanin A

The simplified molecular input line entry specification (SMILES) information of biochanin A was imported into SuperPred (http://prediction.charite.de) [14] to obtain the Anatomical Therapeutic Chemical (ATC) code and the target proteins of biochanin A were predicted by searching the Traditional Chinese Medicine Systems Pharmacology Database and Analysis Platform (TCMSP, http://lsp.nwu.edu.cn/tcmsp.php) [14], STITCH (http://stitch.embl.de) [15], and GeneCards database (https://www.genecards.org/) [16]. Only human target proteins were documented and the information of these targets was downloaded as well.

### 2.2. Potential Targets in Osteosarcoma

All the proteins associated with osteosarcoma were obtained from DisGeNET (http://www.disgenet.org) [17] and the Search Tool for the Retrieval of Interacting Genes (STRING) database (https://string-db.org/) [18]. After amalgamation of the proteins from these two databases, 114 proteins were retrieved from this process. The gather of 114 proteins would be considered as the cluster of potential targets to regulate the biological behavior of osteosarcoma.

### 2.3. Protein-Protein Interaction Data

The protein-protein interaction (PPI) data was obtained from STRING database, which provides information regarding the predicted and experimental interactions of proteins. Furthermore, STRING database defines PPI with confidence ranges for data scores (low confidence: scores <0.4; medium 0.4-0.7; high: >0.7). PPIs with the sum of the scores > 0.7 were picked up for further research in the present study.

### 2.4. Network Construction

Three networks were constructed including biochanin A–targets network, targets of osteosarcoma PPI network, and biochanin A-targets-osteosarcoma network. The biochanin A–targets network was established by linking the biochanin A with its targets retrieved from TCMSP, STITCH, and GeneCards database. Targets of osteosarcoma PPI network were constructed by connecting the proteins which interact with other proteins based on the information of PPI from STRING database and DisGeNET database. On the basis of the previous two networks, the biochanin A–targets-osteosarcoma network was established by reserving the proteins which mediate the biochanin A regulating the biological behavior of osteosarcoma and integrating the PPI of these reserved proteins. The three networks above were visualized by Cytoscape (http://cytoscape.org).

### 2.5. Gene Ontology and Pathway Enrichment

Gene Ontology (GO) and Kyoto Encyclopedia of Genes and Genomes (KEGG) pathway enrichment analysis were performed with the Database for Annotation, Visualization and Integrated Discovery (DAVID, https://david.ncifcrf.gov/, ver. 6.8) [19]. GO terms and pathways with False Discovery Rate (FDR) <0.01 were defined as enriched terms and pathways. The OmicShare tools (http://www.omicshare.com/tools), which are a free online platform for data analysis, were used to chart the bubble plot in KEGG enrichment analysis.

### 2.6. Preparation of 3D Structure Ligands of Biochanin A and Target Protein Activity Pockets

The biochanin A was searched in Pubchem (https://pubchem.ncbi.nlm.nih.gov) to get the 3D molecular structure and saved as “SDF” file format. The target proteins acquired from biochanin A–targets-osteosarcoma network were considered as the key targets in biochanin A treating osteosarcoma. The human structures of these target proteins were collected from the protein data bank (PDB) (http://www.rcsb.org) as potential targets for docking.

### 2.7. Molecular Docking and Comprehensive Score

The whole work of docking was conducted using the commercial software Discovery Studio 2.5 (BIOVIA, San Diego, USA). First, the X-ray crystal structures of protein targets were preprocessed. Hydrogen was added to the model, and its orientation was optimized using the CHARMM (https://www.charmm.org/) force field energy minimization while all nonhydrogen atoms were not allowed to move. Every protein was defined as a receptor, and the proteins' active sites were found from the receptor cavities using the Discovery Studio tool. Then, the docking protocol was performed to show the interaction of components in Discovery Studio with the differential proteins using LibDock. As LibDock can provide 10–100 predicted LibDockscores from different docking poses for each compound in a binding pocket of a protein, we only considered the best LibDockscore. The protein with the highest LibDock score was considered as the target of biochanin A with the most possibility.

### 2.8. Cell Cultures and Cell Viability Assay

Human osteosarcoma cell lines MG63 (ATCC® CRL-1427™) were cultured in DMEM medium supplemented with 10% fetal bovine serum (FBS), 100 U/ml penicillin, and 100 mg/ml streptomycin at 5% CO2, 37°C for incubation. Cells lines were lysed using 0.25% trypsin solutions in combination with 0.02% EDTA and applied for studies when growing to 60-70%. Each group of cells was prepared as monolayer culture and seeded in 96-well plates at 5 × 10^3^ cells/ml. Preincubate the plate for 24 h in a humidified incubator. Add 10 *μ*l of various concentrations of biochanin A (Nature-Standard, Shanghai, China) to the plate at 6 h, 12 h, 24 h, and 48 h, respectively. 10 *μ*l of CCK8 reagent (Beyotime, Nantong, China) was subsequently added to each well for 4 h incubation. The optical density (OD) value was measured at 450 nm in a microplate-reader. The whole process was repeated for three times.

### 2.9. Western-Blot Assay

Total protein from each group was extracted and the concentration was determined using concentration Bradford reagent. Protein samples (100 *μ*l) from each group were obtained and separated by sodium dodecyl sulfate polyacrylamide gel electrophoresis (SDS-PAGE) and transferred on to polyvinylidene fluoride membrane (PVDF). The membrane was then blocked in 5% skim milk solution containing TBST for 1 h at room temperature. Each group of membrane was subsequently treated with dilutions of primary antibody specific for BGLAP (Abcam, Cambridge, USA), ATF3 (Abcam, Cambridge, USA), BAX (Cell Signaling Technology, Danvers, USA), CDKN1A (Cell Signaling Technology, Danvers, USA), and TP53 (Cell Signaling Technology, Danvers, USA) or endogenous reference GAPDH and incubated at 4°C overnight. After that, it was washed for 3 times with TBST and treated with horseradish peroxidase conjugated secondary antibodies for 1-hour incubation at room temperature. The blots were then developed using chemiluminescence for colorimetric detection. The band was scanned for absorbance.

## 3. Results

### 3.1. Biochanin A Target Network

**Figure 1:**
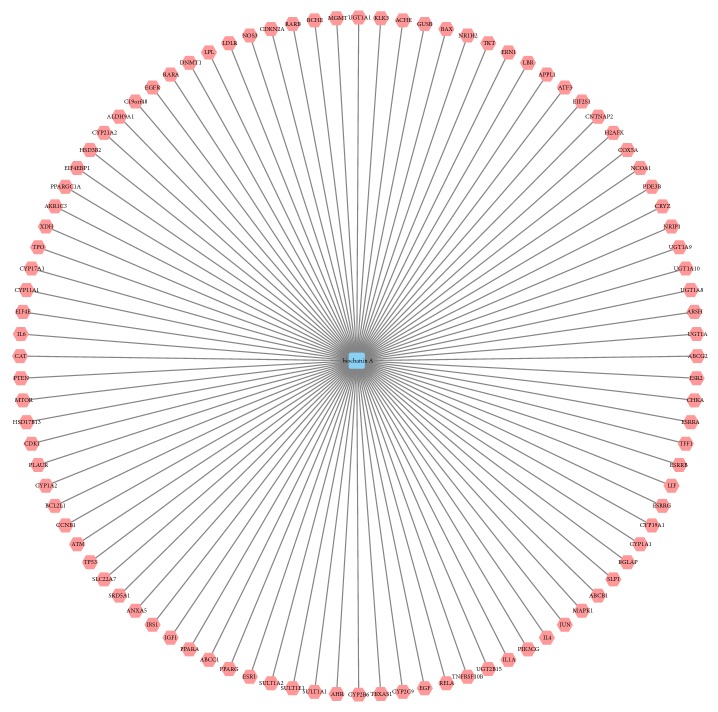
The network shows biochanin A and its 96 targets (pink hexagons represent the target proteins).

As can be seen in Figure 1, ninety-six proteins can be identified as the targets of biochanin A. There are 25 targets identified from TCMSP database, 11 targets identified from STITCH database, and 75 targets identified from GeneCards database, respectively. The details of these 96 target proteins of biochanin A were listed in Table S1. The gather of targets from three databases would be considered as the cluster of biochanin A targeted proteins.

### 3.2. Osteosarcoma Target Network

**Figure 2:**
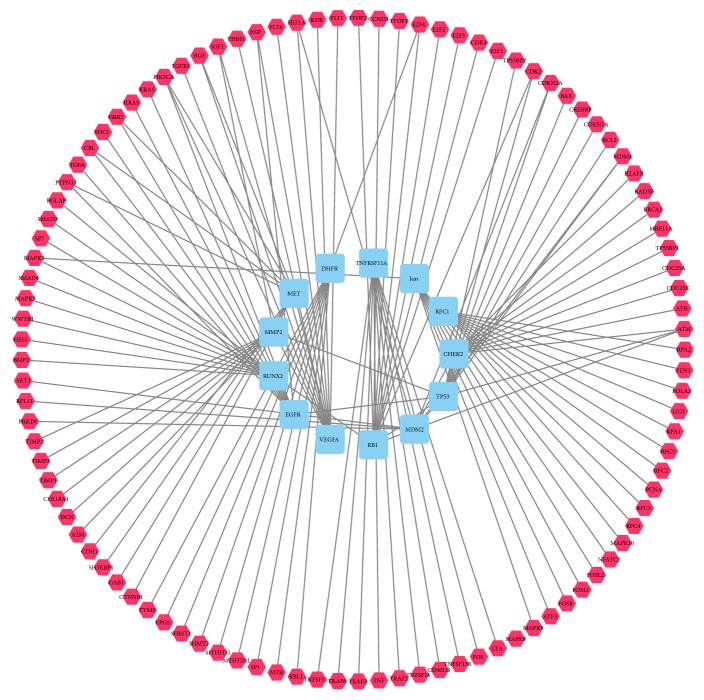
PPI network of osteosarcoma targets (blue squares represent targets related to osteosarcoma; red hexagons represent other human proteins which are directly interacting with the osteosarcoma targets).

By searching the DisGeNET and STRING database, 114 proteins were documented as the disease specific targets of osteosarcoma. These 114 proteins are defined as the osteosarcoma related proteins and the interaction between these proteins were demonstrated in Figure 2. Among these 114 proteins, thirteen proteins, including CHEK2, TP53, RB1, VEGFA, EGFR, RUNX2, MDM2, MMP2, MET, DHFR, TNFRSF11A, JUN, and RFC1, were reported to participate in the pathological process of osteosarcoma directly. The other 101 proteins can regulate the 13 directly related proteins and associate with osteosarcoma indirectly.

### 3.3. PPI Network of Targets for Biochanin A against Osteosarcoma

**Figure 3:**
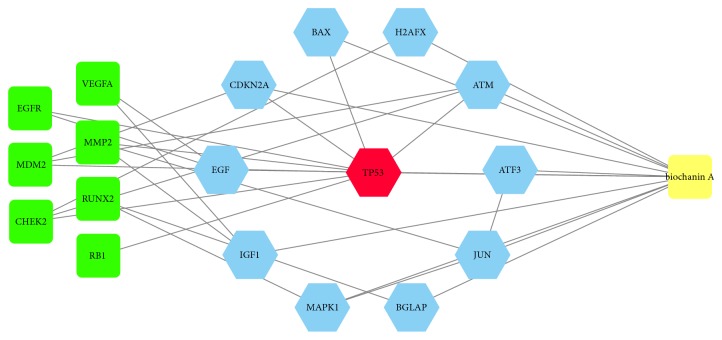
PPI network of targets for biochanin A against osteosarcoma (green squares represent targets related to osteosarcoma and indirectly targeted by biochanin A; blue hexagons represent intersection targets between biochanin A and osteosarcoma targets; red hexagon represents TP53).

To further discover the mechanism of biochanin A against osteosarcoma, the target proteins of osteosarcoma were linked with the target proteins of biochanin A. The PPI network of targets for biochanin A antiosteosarcoma was constructed by integrating Figures 1 and 2 and result was shown as Figure 3. After the integration, ten proteins were reserved as the key targets in biochanin A treating osteosarcoma. These proteins are insulin like growth factor 1 (IGF1), Jun protooncogene (JUN), bone gamma-carboxyglutamate protein (BGLAP), ATM serine/threonine kinase (ATM), mitogen-activated protein kinase 1 (MAPK1), activating transcription factor 3 (ATF3), H2A histone family member X (H2AFX), BCL2 associated X (BAX), cyclin dependent kinase inhibitor 2A (CDKN2A), and epidermal growth factor (EGF), respectively.

### 3.4. Module Analysis

**Figure 4:**
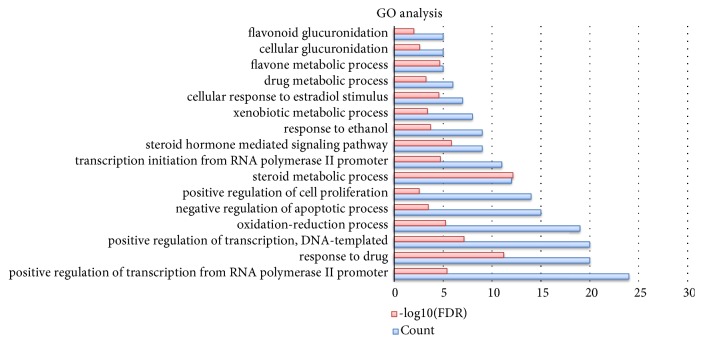
GO enrichment analysis for the targets in biochanin A treating osteosarcoma (p value<0.01 and FDR<0.01). The y-axis shows significantly enriched GO categories of the target genes, and the x-axis shows the enrichment scores of these terms or the counts of targets.

**Figure 5:**
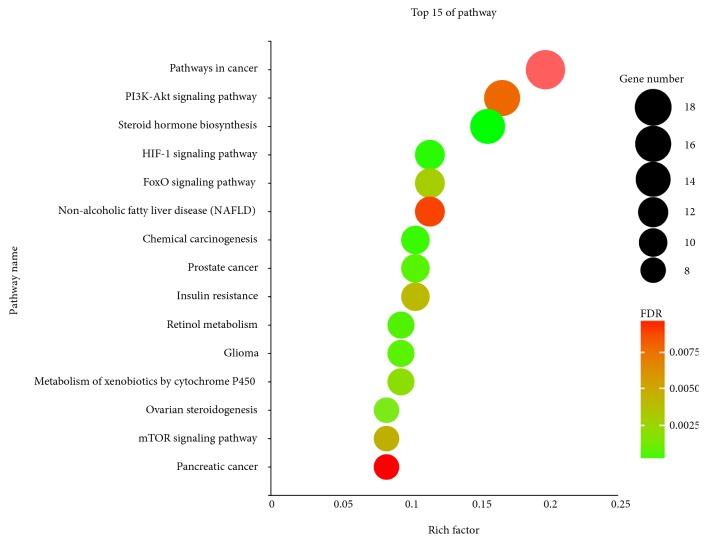
KEGG pathway enrichment analysis for the targets in biochanin A treating osteosarcoma (p value<0.01 and FDR<0.01). The y-axis shows significantly enriched KEGG pathways of the target genes, and the x-axis shows the Rich factor. Rich factor stands for the ratio of the number of target genes belonging to a pathway to the number of all the annotated genes located in the pathway. The higher Rich factor represents the higher level of enrichment. The size of the dot indicates the number of target genes in the pathway, and the color of the dot reflects the different FDR range.

The GO enrichment analysis and KEGG enrichment analysis were also completed to evaluate the module in this study. The GO enrichment analysis plot was shown in Figure 4. It can be found that response to drug, steroid metabolic process, and steroid hormone mediated signaling pathway and DNA-templated positive regulation of transcription are highly associated with this module. As shown in Figure 5, the top 15 of enriched KEGG pathways were displayed. These pathways are mostly related to pathways in different kinds of cancers.

### 3.5. Best Docking Combination to Biochanin A

**Figure 6:**
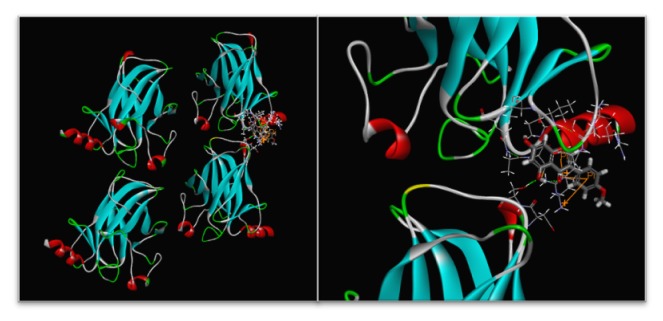
The sketch map of the biochanin A binding to TP53 is showed in the left part of this figure, and the magnified details of the binding were showed in the right part of this figure.

The docking process was performed to screen out the exact targets of biochanin A suppressing osteosarcoma. All the docking sketch maps of target proteins with biochanin A are shown in Figure 6. The LibDock Scoresof the 11 target proteins were shown in Table 1 as well. As we can see from Table 1, TP53 has the highest LibDock score indicating that biochanin A is most likely to bind the TP53 and function as an osteosarcoma repressor. The other proteins may have certain affinity to bind biochanin A, but this affinity is not enough strong to compete with TP53.

### 3.6. Effect of Biochanin A on Cell Viability of MG63

**Figure 7:**
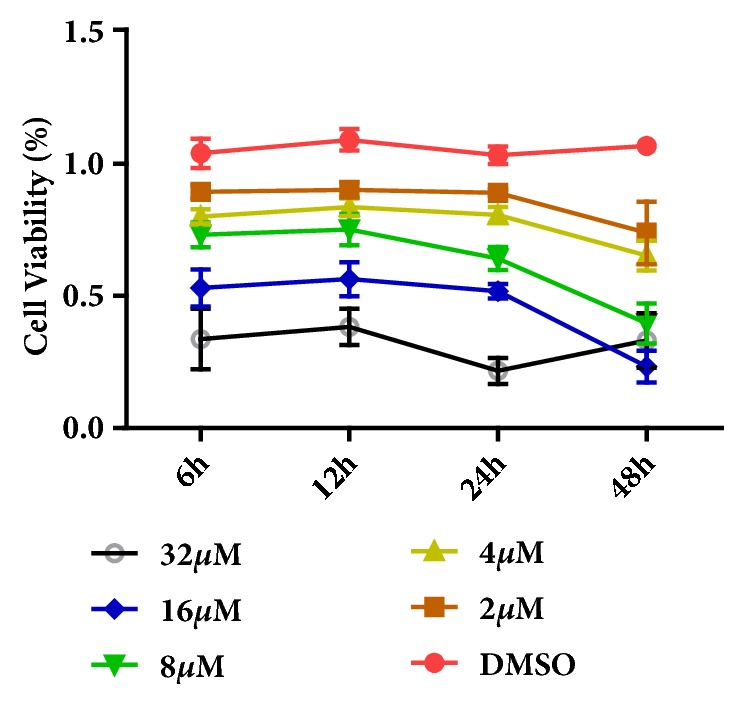
Biochanin A suppressed MG63 cells proliferation significantly under the concentration of 2, 4, 8, 16, and 32 *μ*M with 6 h, 12 h, 24 h, and 48 h incubation, respectively.

To analysis the effect of biochanin A on cell viability of MG63 cells, we treated them with various doses of biochanin A and detected the cell activity using CCK8 technique. The results shown in Figure 7 indicate that, under the concentration of 4 *μ*M, biochanin A could inhibit the MG63 cell survival significantly. According to this result, 4 *μ*M and 8 *μ*M were used for the subsequent experiment.

### 3.7. Effect of Biochanin A on Target Proteins in Osteosarcoma

**Figure 8:**
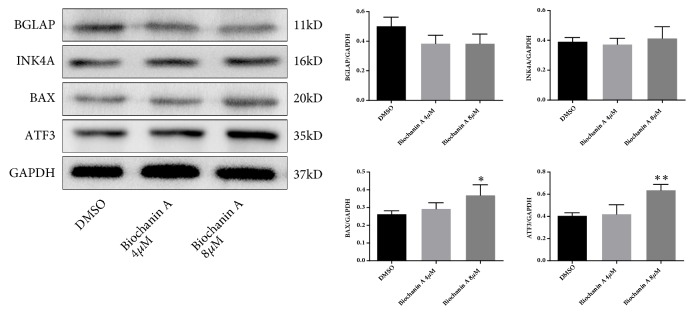
Therapeutical effect of biochanin A on osteosarcoma. Total cellular proteins were obtained from osteosarcoma. BGLAP, INK4A, BAX, and ATF3 were detected using specific antibodies. GAPDH was used as an internal control. Data was presented as mean ± SD from three independent experiments. *∗*p < 0.05; *∗∗*p < 0.01 versus DMSO group.

**Figure 9:**
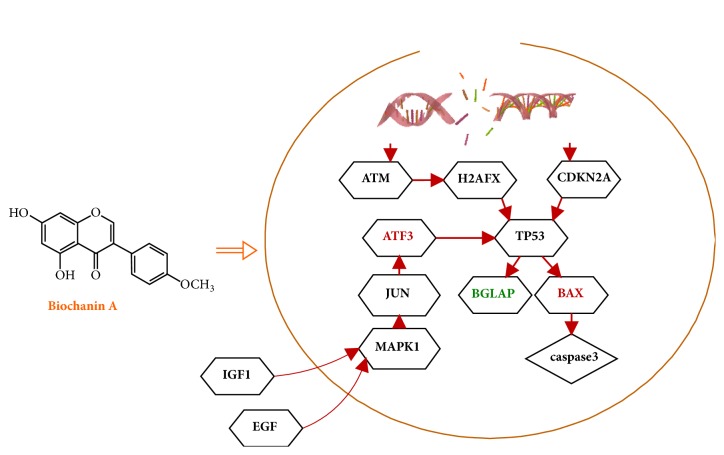
The relationship map of the biochanin A and target proteins. The red arrow represents an activate regulation. Proteins in red indicate that the expression level of the protein is increased, while in green they indicate the expression level of the protein is decreased.

As shown in Figure 8, the expression of BAX and ATF3 was significantly increased after the induction of 8 *μ*M biochanin A in MG63 cells, and the expression of TP53 was decreased. The BGLAP was also repressed by biochanin A, but the result showed no significance. As can be seen in Figure 9, the relationship map of the biochanin A and target proteins was charted based on the results of target proteins' expression.

## 4. Discussion

Osteosarcoma is the most common malignant tumor in orthopedics with poor prognosis and high mortality [9]. Because of the early metastasis of osteosarcoma, surgical treatment was too late to eliminate all the sarcoma cells [10, 12]. Therefore, chemotherapy is an irreplaceable therapy which significantly prolongs the survival of osteosarcoma patients. In view of the insufficient effect, considerable side effects, and chemoresistance of chemotherapeutic drugs, it is of great value to discover a new effective drug to improve the chemotherapy of osteosarcoma.

Biochanin A is a natural compound found in Trifolium pratense L and can work as phytoestrogen in some physiological and pathologic process in vivo [3]. Unlike some other phytoestrogen's facilitating roles in gynaecologic cancer, biochanin A has an antitumor effect on pharynx squamous carcinoma, hepatocellular carcinoma, pancreatic cancer, prostate cancer, and colon tumor in addition [1, 2, 5–8]. As a result, biochanin A may inhibit cancer in other mechanisms. Recently, a study has demonstrated that biochanin A can suppress the proliferation of osteosarcoma by arresting the cells in G0/G1 stage, but the underneath mechanisms were not discussed [11].

In present study, network pharmacology was employed to dock the targets of biochanin A with the important functional signal nodes of osteosarcoma. By searching the TCMSP, STITCH, and GeneCards database, 96 potential targets of biochanin A were identified. At the same time, 114 target proteins from DisGeNET database and STRING database were defined as the key nodes in regulating the osteosarcoma. In these proteins, there were 11 intersection proteins between biochanin A targets and osteosarcoma related targets, namely, TP53, IGF1, JUN, BGLAP, ATM, MAPK1, ATF3, H2AFX, BAX, CDKN2A, and EGF.

After the bioinformatics analysis and reviewing the relate articles, interestingly, we found that most of these 11 intermediate target proteins have a close relationship with the double strand DNA break (DSB). DSB self-surveillance escaping is a main mechanism in the development of osteosarcoma that cancer cells can neglect the DSB self-surveillance escaping duplicates with DNA damage [20]. As a result, errors in DNA accumulate and more oncogenic variation of gene will appear to promote the proliferation of osteosarcoma.

TP53, also called p53, is a core protein functioning in the DNA damage self-surveillance, which can arrest the cell in G1 phase if there is a defect in DNA completeness and continuity [20]. As one of the key signaling pathway, the RAS/ERK/c-JUN signaling pathway plays a key role in the downstream regulation of DSB. JUN and MAPK1 are the main components of this pathway. When the DSB occurs, ATM will be recruited and phosphorylate the H2AFX on Ser 139, which is the earliest response to DSB [21, 22]. The phosphorylated H2AFX stabilizes the binding of TP53 binding protein (P53BP1) with DSB [23]. Then the P53BP1 binds to the core region of TP53 with BRCA1 C-terminus (BRCT) domain in tandem with carboxyl terminal of P53BP1, activating the transcription of TP53 [23]. CDKN2A is also a biomarker of DSB, which elevates after DNA damage [24]. CDKN2A also plays a role as TP53 stabilizer [25]. CDKN2A can bind to MDM2 and TP53 and inhibits MDM2's TP53 degradative function. Lack or inactivate of CDKN2A may lead to decreasing level of TP53. Other oncogenic signaling pathways are also involved in the effect of biochanin A on osteosarcoma. Both IGF1 and EGF have a growth hormone-like function [26, 27]. They can bind to their receptor and active downstream MAPK signaling pathway [28]. TP53 could be upregulated by this pathway and MAPK1 and JUN proteins play a role as key signal nodes in this pathway as well [29]. But by testing the concentration of these proteins, we did not observe the obvious changes in the expression of TP53, IGF1, JUN, ATM, MAPK1, H2AFX, CDKN2A, and EGF.

BGLAP, also known as osteocalcin, is a small and highly conserved protein, which was identified in the mineralized matrix of bone [30]. In previous study, expression of BGLAP can be upregulated by the activation of the TP53 and CDKN2A signaling pathway [31]. Han et al. have demonstrated that, as a biomarker of bone, the expression of BGLAP was decreased in dying or undifferentiated MG63 cells [32]. In this study, after the treatment of biochanin A, a decreasing trend of BGLAP level can be seen from the western-blot, which may also indicate the death of osteosarcoma cells.

ATF3 was found to mediate anticancer activity in several malignant tumors [33, 34]. It can also activate MAPK signaling pathway and increasing the expressing of TP53 [35]. Our research highlighted that biochanin A treatment can lead to an increasing of ATF3. Combined with previous researches, biochanin A may play an antitumor role in osteosarcoma by the ATF3 pathway.

BAX, essential in the release of caspase 3, was reported with a strong connection with osteosarcoma [36]. Apoptosis of cancer cells induced by TP53 needs to be mediated by BAX [37, 38]. Zhao et al. have mentioned that biochanin A can contributed to the increasing of BAX in osteosarcoma. In this study, we also found that the expression of BAX was increased after the application of biochanin A, which is consisted with previous studies.

Molecular docking is the most widely used method for calculating protein-ligand interactions, and we used the LibDock program in Discovery Studio 2.5 software to investigate the probable binding modes. The results show that biochanin A has a high affinity for TP53. Combining the results in this study with the network pharmacological analysis, it can be seen from figure 9 that the biochanin A has an antiosteosarcoma effect and may associate with DSB response, which may also partly explain why the osteosarcoma cells were arrested in G0/G1 phase in the previous report. But the effect of biochanin A on TP53 was not discussed in this study, and more researches are needed to unveil whether biochanin A can bind to TP53 and the expression of BGLAP, BAX, and ATF3 was regulated through TP53 pathway.

## 5. Conclusion

In present study, we predicted and concluded the mechanism of biochanin A treating osteosarcoma by using the network pharmacology approach. Based on the analysis above, we supposed that biochanin A can suppress the proliferation of osteosarcoma and regulate the expression of BGLAP, BAX, and ATF3, which are involved in regulating DSB response, differentiation and cell death of osteosarcoma. Present study can promote our understanding and provide a new sight for the researchers to further demonstrate the mechanism of biochanin A treating osteosarcoma.

## Figures and Tables

**Table 1:** The docking score of each target protein with biochanin A.

protein	LibDock Score
ATM	72.042
TP53	106.505
H2AFX	N/A
BAX	69.179
CDKN2A	N/A
EGF	79.287
IGF1	77.817
MAPK1	90.931
JUN	78.29
ATF3	67.019
BGLAP	N/A

## Data Availability

All the data used to support the findings of this study are included within the article and its supplementary information file.

## Supplementary Materials

Supplementary Materials Table S1: target protein of biochanin A in homo sapiens. Table S2: target protein associated with the pathogenesis of osteosarcoma.
