# Supplementary material for: Network Pharmacology Integrated Molecular Docking Reveals the Antiosteosarcoma Mechanism of Biochanin A
Source: Evid Based Complement Alternat Med. 2019 Jan 6;2019:1410495. doi: 10.1155/2019/1410495 (PMC6339762; doi:10.1155/2019/1410495)
Supplement: Supplementary Materials — Table S1: target protein of biochanin A in homo sapiens. Table S2: target protein associated with the pathogenesis of osteosarcoma. [file 1410495.f1.docx]

| Name of proteins | | | | | | | | | |
| --- | --- | --- | --- | --- | --- | --- | --- | --- | --- |
| ABCG2 | ESR2 | CHKA | ESRRA | TFF1 | ESRRB | LIF | ESRRG | CYP19A1 | CYP1A1 |
| BGLAP | SLPI | ABCB1 | MAPK1 | JUN | IL4 | PIK3CG | IL1A | UGT2B15 | TNFRSF10B |
| RELA | EGF | CYP2C9 | TBXAS1 | CYP2B6 | AHR | SULT1A1 | SULT1E1 | SULT1A2 | ESR1 |
| PPARG | ABCC1 | PPARA | IGF1 | IRS1 | ANXA5 | SRD5A1 | SLC22A7 | TP53 | ATM |
| CCNB1 | BCL2L1 | CYP1A2 | PLAUR | CDK1 | HSD17B13 | MTOR | PTEN | CAT | IL6 |
| EIF4E | CYP11A1 | CYP17A1 | TPO | XDH | AKR1C3 | PPARGC1A | EIF4EBP1 | HSD3B2 | CYP21A2 |
| ALDH9A1 | C19orf48 | EGFR | RARA | DNMT1 | LPL | LDLR | NOS3 | CDKN2A | RARB |
| BCHE | MGMT | UGT1A1 | KLK3 | ACHE | GUSB | BAX | NR1H2 | TKT | ERN1 |
| LBR | APPL1 | ATF3 | EIF2S1 | CNTNAP2 | H2AFX | COX5A | NCOA1 | PDE3B | CRYZ |
| NRIP1 | UGT1A9 | UGT1A10 | UGT1A8 | ARSH | UGT1A |  |  |  |  |

Table S1 Target protein of biochanin A in homo sapiens.

Table S2 Target protein associated with the pathogenesis of osteosarcoma.

| Name of proteins | | | | | | | | | |
| --- | --- | --- | --- | --- | --- | --- | --- | --- | --- |
| CHEK2 | TP53 | RB1 | VEGFA | EGFR | RUNX2 | MDM2 | MMP2 | MET | DHFR |
| TNFRSF11A | JUN | RFC1 | ATM | ATR | CDC25C | CDC25A | TP53BP1 | MRE11A | BRCA1 |
| RAD50 | H2AFX | MDM4 | BCL2 | CDKN1A | CREBBP | BAX | CDKN2A | CDK2 | TP53BP2 |
| E2F1 | CDK4 | E2F3 | E2F2 | E2F4 | TFDP1 | CCND1 | TFDP2 | FLT1 | KDR |
| HIF1A | FLT4 | EGF | THBS1 | IGF1 | HGF | TGFB1 | PIK3CA | KRAS | HRAS |
| GRB2 | SHC1 | CBL | TGFA | PTPN11 | BGLAP | SMAD3 | SP7 | MAPK1 | SMAD4 |
| MAPK3 | WWTR1 | HES1 | BMP2 | AKT1 | RPL11 | PRKDC | TIMP2 | TIMP3 | TIMP1 |
| COL18A1 | DCN | A2M | CDH1 | SH3KBP1 | GAB1 | CTNNB1 | TYMS | FPGS | SHMT1 |
| SHMT2 | MTHFD1 | MTHFD1L | SP1 | MTR | RBL1 | TNFSF11 | TRAF6 | TRAF3 | TNF |
| TRAF2 | TNFSF14 | CD40LG | TNFSF13B | FOS | LTA | MAPK8 | MAPK9 | ATF3 | FOSB |
| FOSL1 | FOSL2 | NFATC2 | MAPK10 | RFC4 | RFC3 | PCNA | RFC2 | RFC5 | RPA1 |
| LIG1 | POLA2 | FEN1 | RPA2 |  |  |  |  |  |  |
